# A Review of the Effect of Management Practices on *Campylobacter* Prevalence in Poultry Farms

**DOI:** 10.3389/fmicb.2018.02002

**Published:** 2018-08-24

**Authors:** Nompilo Sibanda, Aaron McKenna, Anne Richmond, Steven C. Ricke, Todd Callaway, Alexandros Ch. Stratakos, Ozan Gundogdu, Nicolae Corcionivoschi

**Affiliations:** ^1^School of Biological Sciences, Queen’s University Belfast, Belfast, United Kingdom; ^2^Moy Park, Ltd., Craigavon, United Kingdom; ^3^Center for Food Safety, Department of Food Science, University of Arkansas, Fayetteville, AR, United States; ^4^Department of Animal and Dairy Science, University of Georgia, Athens, GA, United States; ^5^Bacteriology Branch, Veterinary Sciences Division, Agri-Food and Biosciences Institute, Belfast, United Kingdom; ^6^London School of Hygiene and Tropical Medicine, London, United Kingdom

**Keywords:** *Campylobacter*, poultry, farm practices, biosecurity, management

## Abstract

Poultry is frequently associated with campylobacteriosis in humans, with *Campylobacter jejuni* being the most usual *Campylobacter* associated with disease in humans. Far-reaching research on *Campylobacter* was undertaken over the past two decades. This has resulted in interventions being put in place on farms and in processing plants. Despite these interventions, coupled with increased media coverage to educate the consumer on *Campylobacter* prevalence and campylobacteriosis, human health incidents are still high. Recent research is now shifting toward further understanding of the microorganisms to challenge interventions in place and to look at further and more relevant interventions for the reduction in human incidents. Farm practices play a key role in the control of colonization within poultry houses and among flocks. Prevalence at the farm level can be up to 100% and time of colonization may vary widely between flocks. Considerable research has been performed to understand how farm management and animal health practices can affect colonization on farms. This review will focus on farm practices to date as a baseline for future interventions as the microorganism becomes better understood. Further research is required to understand the chicken microbiome and factors influencing vertical transmission. The persistence of *Campylobacter* in animal and environmental reservoirs within and around farms requires further investigation to tailor farm practices toward preventing such reservoirs.

**IMPLICATIONS**

This review gives an overview of farm practices and their effect on *Campylobacter* prevalence in poultry. Various elements of farm practices have been captured in this review.

## Introduction

*Campylobacter*, as a genus are corkscrew shaped and motile Gram negative microorganisms with an optimal growth at 42°C. They are generally isolated from the intestines of cattle, sheep, swine, and the poultry caecum. Because of a higher body temperature poultry and other avian species are the most common food animal that hosts for *Campylobacter* spp., representing the main source of infection for humans (Silva et al., 2011). While three *Campylobacter* species (*C. jejuni, C. coli*, and *C. lari*) are related to the poultry digestive system and to foodborne infections, *Campylobacter jejuni* is considered the dominant specie in relation to its impact on human health (Shane, 1992; Corcionivoschi et al., 2012; Cean et al., 2015; Ugarte-Ruiz et al., 2018). A limited number of studies have reported possible negative health implications in chickens caused by *C. jejuni* colonization of the gut, therefore this bacterium is considered to have a near-commensal relationship with the chicken (Thibodeau et al., 2015). Only a few *Campylobacter* cells from undercooked or raw chicken are able to cause human illness (Bhaduri and Cottrell, 2004) and over 100 deaths in the United Kingdom annually, with an estimated cost of £1 billion to the United Kingdom National Health Service (Tam and O’Brien, 2016). Most human cases of campylobacteriosis in the United Kingdom are attributed to contaminated poultry (Skarp et al., 2016; Lee et al., 2017; Oh et al., 2017). However, Lynch et al. (2011), reported that most *Campylobacter* in their study were isolated from beef (36% prevalence), pork (22%), and chicken (16%). *Campylobacter* has also been found in unpasteurised milk and untreated water. Because of the common nature of this pathogenic threat, a broad-based approach in all segments of the food industry has been taken in the United Kingdom to reduce incidents of campylobacteriosis. Efforts have been made across the supply chain; including farms, processing plants, retailers, and through educating the public. Rejection of poultry carcasses at the slaughter for pathological reasons was it has been reported to be certainly associated also with *Campylobacter* presence (Powell et al., 2012). Collectively, results suggest that *Campylobacter* is often present in broilers in a poor health condition, therefore suggesting that the mortality rate could be an efficiency marker for farm management practices and biosecurity. However, because there is not a linear trend in the data, it suggests that when greater mortality rates are recorded, then antibiotics are prescribed which impacts upon *Campylobacter* colonization and or contamination.

Powell et al. (2012) surveyed 25 United Kingdom abattoirs which represented 88% of the total United Kingdom poultry production, reported that 75% of chicken carcasses were contaminated with *Campylobacter* from feces, and that 87% of this contamination occurred at slaughter. In the most recent survey by Public Health England (2015), the prevalence of *Campylobacter* spp. In the United Kingdom, within fresh chicken at the retail level was 73.3%. A significant proportion (19.4%) of the positive chicken carcasses had levels of >1000 CFU/g of chicken skin. Also, a higher percentage of positive chickens were contaminated with higher levels of *Campylobacter* spp. during the summer compared to winter period. Speciation revealed that *C. jejuni* was identified in most of the positive chicken skin samples (76.6%) whereas *C. coli* was present in only 13.9% of samples. It was suggested by Powell et al. (2012) that a 2-log reduction in the number of *Campylobacter* on chicken carcasses would lead to a 30-fold drop in human cases connected to contaminated chicken meat. *Campylobacter* are able to survive in different farm environments and have been found in the air, soil, water, dust, and abiotic surfaces (Bull et al., 2006; Ellis-Iversen et al., 2009).

Most transmission to poultry occurs from the environment, as well as through horizontal transmission between flock mates, but once *Campylobacter* colonizes a broiler flock, the spread is fast making an eradication approach impossible. A well-designed and planned biosecurity approach at farm level have been established as a fundamental method to counteract colonization of flocks (Georgiev et al., 2017). The present review will focus on the avian management practices on poultry farms and what effect these have on *Campylobacter* prevalence.

## Biosecurity

Biosecurity is an important measure for the control of *Campylobacter* because once colonization occurs in a poultry flock the horizontal transmission can be rapid (Battersby et al., 2016). A study by Gibbens et al. (2001) demonstrated that well-implemented disinfection protocols could lead to a decrease in *Campylobacter* prevalence from 80 to less than 40% in broilers (Gibbens et al., 2001). Installing hygienic barriers between internal and external environments, controlling the entry of personnel, strict hygiene rules (handwashing and sanitizing hands), changing boots and overalls before entering have been shown to be effective (Silva et al., 2011). A literature review conducted from 1980 to 2008 concluded that high standards in biosecurity measures should contribute to the reduction of flock *Campylobacter* colonization (Newell and Fearnley, 2003). Risk factors with respect to increased *Campylobacter* colonization include: deprived farm hygiene, a reduced flock replacement period, the presence of other farm animals, rodents and insects, seasonal changes and partial depopulation (Russa et al., 2005). Hygiene and physical barriers are specific biosecurity measures which should be efficient in protecting poultry against *Campylobacter*. Increased biosecurity measures are always associated with absence of *Campylobacter* in poultry however, the impact is difficult to measure (Lin, 2009). Controlling *Campylobacter* on the farm is complex as the pathogen can persist in a variety of environments and hosts that can be found on the farm (Lynch et al., 2011).

*Campylobacter* can be carried into the broiler house via boots, clothes and equipment. The use of dedicated footwear has proven to be the most important risk factor in a Danish study (Newell and Fearnley, 2003). Other factors that affected biosecurity measures were the greater number of people working on the farm increased the chances of biosecurity breaches (Newell et al., 2011). Biosecurity measures are to include equipment such as sanitizing of buckets used to lift dead birds and any equipment brought into the house (Newell and Fearnley, 2003). Hygiene measures by on farm personnel include handwashing, use of separate boots for each house, use of footbath disinfectants, limited access to key personnel only, pest control and staff training (Sahin et al., 2015). The entrance of the catching crew and catching equipment is often a serious breach of biosecurity because external sources posing a 21% contamination rate, this is a vehicle for introducing contamination, particularly during depopulation (Ellis-Iversen et al., 2012). Farm emergency procedures should also consider the biosecurity measures to ensure these are adhered consistently. Staff training is key to the successful implementation of these biosecurity measures at all times, but is most especially impactful in an emergency (Sahin et al., 2015). An observational study in Canada reported that an average of four biosecurity breaches occurred per farm visit. Out of all the breaches, 61% of faults were linked to cross contamination of clean and contaminated zones, 14% related to improper use of footwear, 11% inadequate hand washing, and 7% unclean overalls. This high error rate may be attributed to lack of training or lack of understanding of consequences amongst other reasons (Battersby et al., 2016).

The efficient use of boot baths increased the interval to infection considerably. The presence of boot baths at the entrance of the broiler house is considered a risk factor (Borck Hog et al., 2016) and for these to be efficient, high maintenance with disinfection replenishment being done at regular intervals. If not well-maintained the footbaths will increase the risk rather than act as a defensive barrier. Disinfectants should be replaced weekly or if the dilution is reduced (due to rain water) or if there is organic matter build up at the bottom of the foot bath, they must be replenished (Evans and Sayers, 2000).

Strict biosecurity and farm hygiene measures have been shown to be linked with reduced *Campylobacter* positive flocks (Newell et al., 2011). However, the most severe biosecurity controls are not most efficient in preventing the presence of *Campylobacter* (Russa et al., 2005) because they are dependent on personnel following the correct protocols at all times. Even when the most stringent controls are followed, there may still be contamination of door handles and other contact areas (fomites) that are often misunderstood. This may result in the contamination of a new flock during chick placement, which ensures that the pathogen has a long time to spread horizontally within a new “clean” flock during the grow out phase. Biosecurity measures are also not always cheap to implement, and the purchase cost may be prohibitive. This includes both the cost of equipment to have the biosecurity barriers as well as the cost of time for farm personnel in implementing these measures. On evaluating the cost effectiveness of *Campylobacter* interventions, van Wagenberg et al. (2016) concluded that the most cost effective procedure was to apply barriers in each house and to utilize dedicated tools for each house to minimize the cross-contamination risk; however, the most cost effective intervention was the ban of partial depopulation and introducing the all-in/all-out system at approximately day 35. Hygiene barriers proved effective to some extent in preventing *Campylobacter* infection in broiler flocks. These results suggested that expanding the hygiene barriers to include gates, vehicle disinfection, respecting biosecurity measures during catching would increase *Campylobacter* reduction, however the value (cost effectiveness) of these additional efforts would not be detectable in the final results (Hald et al., 2000). Preventing farm staff from direct contact with the broilers has been shown to protect the broilers from *Campylobacter* infection (Battersby et al., 2016). A study by Ridley et al. (2011) found *Campylobacter* matching the subsequent flock type found in the catcher lunch bags, further emphasizing the risk of employees bringing personal items onto the farm (Ridley et al., 2011).

Harmonization, benchmarking, and implementation of universal controls for biosecurity measures across different countries is difficult due to the different farm practices, housing, production stages, and equipment designs (Sahin et al., 2015). A study conducted on Danish farms concluded that factors like the oldness of the poultry house, rodent control, the age of broiler at slaughter, storage of whole wheat, number of chimneys on the broiler house, the location of the broiler farm in relation to cattle density are very important in controlling *Campylobacter* presence. However, it must also be taken in account that these observations are specific to Danish farm practices and may differ from one country to the next, however, they are a valuable place to begin understanding the value of proper biosecurity appreciation and implementation, as well as some of the challenges that underpin implementation on every farm world-wide (Sommer et al., 2013).

Ridley et al. (2011) carried out a study to compare *Campylobacter* prevalence in normal farm practices versus enhanced biosecurity farm practices. Findings revealed that although there was a reduction in *Campylobacter* presence with enhanced biosecurity measures (up to 35% reduction in vehicles), the enhanced biosecurity measures were insufficient to prevent flock colonization. Unfortunately, once a flock is infected, it is very difficult to eliminate *Campylobacter* in a flock, because chickens (along with other species of animals) are carriers and major reservoirs of *Campylobacter*. They act as passive transmitters and amplifiers via the spread of fecal contamination. Following the entry of *Campylobacter* into a newly occupied house all the birds can become positive within a week based on cloacae swabs, demonstrating that *Campylobacter* colonization spreads very rapidly amongst housed broiler chickens (Evans and Sayers, 2000). Results of a questionnaire study by Hald et al. (2000) indicated that other animals located in the intermediate vicinity of the broiler house posed a significant risk to broiler flocks in terms of *Campylobacter* colonization. It is strongly suggested that a farmer tending both cattle and poultry on the same farm transmitted *Campylobacter* from cattle to poultry (and vice versa) on his/her boots (van de Giessen et al., 1998) Farm personnel and equipment (e.g., feed trucks) can carry *Campylobacter* between broiler houses and onto subsequent or neighboring farms (Newell et al., 2011). In the absence of infected neighbors in 2 km radius of susceptible farm, in the same month, showed a significant protective effect in comparison with presence of infected neighbors in the same distance and time (Chowdhury et al., 2012). Livestock and broiler farms with flocks positive for *Campylobacter* spp. within a few kilometers distance, as well as heavy rainfall events constitute significant risks for colonization in broiler flocks (Jonsson et al., 2012). Rainfall events may also result in unintentional and unrecognized breaches of biosecurity measures, increasing the routes by which *Campylobacter* can colonize broiler flocks.

## Partial Depopulation

Thinning of a flock is a significant threat to biosecurity and increases the risk of *Campylobacter* contamination by compromising isolation of birds that are not depopulated within a house or a flock. Most of previously declared as *Campylobacter* free broilers can be rapidly contaminated during the process of thinning (Sahin et al., 2015). A study conducted in Ireland revealed that 85% of flocks were positive at depopulation, and their results identified thinning as a significant risk factor for *Campylobacter* introduction and the authors provided the suggestion was that partial depopulation should be discontinued (Smith et al., 2016). The importance of thinning was also emphasized in a trial conducted by Ellis-Iversen et al. (2012) which showed that 83% of the *Campylobacter* positive flocks are associated with this process of partial depopulation and only 43% are identified as positive in the absence of thinning. Age of birds and depopulation are closely associated, making it difficult to be certain which of these two factors affects *Campylobacter* prevalence most significantly, but more recently it has been shown that seasonality is also an important factor on the prevalence that *Campylobacter* in broiler flocks that have not been thinned (Jorgensen et al., 2011). Russa et al. (2005) suggested that there was a link between age at depopulation and *Campylobacter* prevalence. Although the method used in their investigation is not clear, the results show that the proportion of *Campylobacter* positive flocks increased with increasing age. They also detected a link between the proportion of *Campylobacter* positive flocks to weather where higher numbers were seen in the autumn (Russa et al., 2005). Live bird crates that were contaminated with *Campylobacter* from previous (or other) flocks are reintroduced on the farm during catching, and quite often these crates undergo inadequate washing at the slaughter house (Newell et al., 2011). Crates can carry identical genotypes of microorganisms which originate from broiler flocks and abattoirs, which suggests that transport crates are responsible for contamination during transport to slaughter or they could contribute to the *Campylobacter* colonization of broiler houses (Hastings et al., 2011). Research has shown that *Campylobacter* can survive on crates post-sanitization (Hansson et al., 2005; Allen et al., 2008). Results from the survey by Powell et al. (2012) showed company specific risk factors or probable recurrence of strains within a company, this warrants further investigation.

## Cleaning and Disinfection

Due to the intensive cleaning and disinfection that is often between flocks it is difficult to predict *Campylobacter* infection from the status of previous flocks. When farms remove litter between grow-out periods, it is often found that negative flocks follow positive ones. The presence of colonized flocks was linked to the turnaround time in a house. Periods of over 14 days can decrease the possibility of residual bacterial contamination (Newell et al., 2011). The benefit of longer turnaround periods is also supported by Battersby et al. (2016) who state that rapid flock turnover contributes to *Campylobacter* carry over with increased risk being reported if houses are restocked within 9 days of depopulation. A study by Jonsson et al. (2012) also investigated the effect of the length of time the house was empty. Based on a small data set, the study showed that keeping the broiler house empty for less than 9 days would increase the risk for *Campylobacter* spp. Also, if the empty time is extended the risk of introducing *Campylobacter* into the houses is kept low only if the biosecurity and hygiene levels are maintained optimal (Borck Hog et al., 2016). It is well-known that an external reservoir can host multiple *Campylobacter* strains, during the empty period, which will allow colonization of the new flock (Ellis-Iversen et al., 2012).

Using real time PCR Battersby et al. (2016) first detected *Campylobacter* before chick arrival in both internal and external broiler house samples but many of the flocks were negative for *Campylobacter* prior to slaughter. These results indicate that only *Campylobacter* DNA was detected or *Campylobacter* were present in a viable but non-culturable (VBNC) state. In this VBNC state *Campylobacter* can survive for at least 7 months (Lázaro et al., 1999). Further research is required to understand the role of VBNC cells in *Campylobacter* cross contamination.

When looking at persistent external reservoirs, Ellis-Iversen et al. (2012) found that contaminated shed entrances, anterooms and drinkers and shedding of *Campylobacter* by other animals (e.g., cattle, dogs, rodents) have been found to be linked to positive flocks. In order to reduce the risk of *Campylobacter* introduction into the shed they have suggested disinfecting the surroundings of the poultry shed around day 25 of the cycle. Other reservoirs of contamination include, vehicles, equipment used by catchers and catching crews (Ellis-Iversen et al., 2012).

## Antibiotic Usage

Antibiotics are widely utilized in poultry production around the world to improve production efficiency, but they can also impact the microbial population of the gut, including populations of pathogenic bacteria. Campylobacteriosis is a zoonotic foodborne illness, therefore presence of antibiotic resistant *Campylobacter* strains could also impact on human health. It has been indicated that the usage of fluoroquinolones in poultry farms is associated with increased resistance in chicken and human *Campylobacter* isolates (Wieczorek and Osek, 2013).

Most campylobacteriosis patients will not require specific treatment other than fluid replacements but there are situations in which antibiotics, such as tetracycline, fluoroquinolones were used (Silva et al., 2011). The resistance of *Campylobacter* strains to these antibiotics compromises the effectiveness of human treatments. In countries where the use of antibiotics in broiler production well-controlled it is unlikely to have a high prevalence of resistant strains (Norstrom et al., 2007). However, it has been shown that the use of these antibiotics as the first line of treatment in humans led to the development of significant resistance and their efficacy should be re-analyzed (Shobo et al., 2016).

In the E.U. in 2013, it has been reported that ciprofloxacin resistance among human *Campylobacter* isolates ranged from 23% in Denmark to 92%, in Spain, however, the resistance among broiler isolates ranged from 0% in Finland to 90% in Spain (European Food Safety Authority and European Centre for Disease Prevention and Control, 2015). A promising strategy in reducing *Campylobacter*, at gut level, could be achieved by the supplementation with additives in feed that could reduce pathogen caecal colonization by exacting an effect on *Campylobacter* themselves or by altering the chicken caecal microbiome toward a composition that will not favor *Campylobacter* growth and/or survival. Essential oils and organic acids are known as effective in reducing the colonization levels in broiler chickens. According to Grilli et al. (2013) a blend of propionic and sorbic acid, and eugenol and thymol significantly reduced *C. jejuni* in slaughter-age chickens even at low doses (0.1%). Hermans et al. (2010) also investigated the effect of caproic, caprylic, and capric acids against *C. jejuni.* Although, *in vitro* results showed promising antimicrobial activity, *in vivo* testing showed that there was not a reduction in *C. jejuni* when they were included in the feed, 3 days before they were sacrificed. Thibodeau et al. (2015) investigated the chicken caecal microbiome to establish the effect of a non-antibiotic feed additive (mixture of short chain organic acids and phenolic essential oils on *C. jejuni* colonization). Their study concluded that the microbiome is not extensively disturbed when colonized by *C. jejuni.* The investigation also concluded that the feed additive used could significantly reduce *C. jejuni* colonization without significantly affecting the gut microbiome of the chicken.

## Seasonality of Infections or Carriage

The public health burden due to campylobacteriosis cases necessitates the characterization of the seasonal patterns of *Campylobacter* contamination since the possibility of illness increases with increasing levels of contamination. The prevalence of *Campylobacter* in chickens has been found to be associated with seasonality (Taylor et al., 2013; Friedrich et al., 2016). In western countries with temperate climates seasonal peaks of human campylobacteriosis are observed between July and August. The summer peaks in human infection are consistent with higher *Campylobacter* isolation levels from chickens in the summer period, compared to winter, with the human infection peaks preceding the chicken one suggesting a link between the two (Skarp et al., 2016). The reasons behind *Campylobacter* seasonality are not clear yet. However, an increase in pathogen reservoirs, changes in human behavior and climate can influence the shedding and transmission of the pathogen. There is a clear risk level of acquiring campylobacteriosis between rural and urban regions and this risk must be taken in consideration (Deckert et al., 2014; Williams et al., 2015).

Research has also shown that the sources of environmental exposure are season dependent with flies being a common vehicle of transmission between the environment and food (Ekdahl et al., 2005). The use of fly screens ventilation openings was recently described as an efficient method to reduce the number of *Campylobacter* positive flocks (Sahin et al., 2015). These findings confirm that flies serve as a vector particularly during the summer months when temperatures are high (Sahin et al., 2015). In a study conducted in Denmark, the use of fly screens reduced the prevalence of *Campylobacter* from 41.4 to 10.3% in 10 farms. A cluster of farms within 4 km from contaminated flocks were also positive. This is believed to be due to carriers such as flies, however, flies caught from four ante-rooms in houses with *Campylobacter* free chickens were always negative (Berndtson et al., 1996). Four fly samples from positive flocks in the same study were positive. *Campylobacter* spp., have been isolated from beetles originating form poultry farms (Hald et al., 2000). The isolation of *Campylobacter* from the small intestine and the caeca also exhibited a seasonal pattern with a specific increase during the summer months. This pattern was also confirmed in a review of performed over a period of 10 years in Europe in six countries (Jore et al., 2010). Approximately, 2.1–3.5% of annual human campylobacteriosis are associated with wild birds or wild bird infected sources (Cody et al., 2015). This issue is also very much related to seasonality as it has been shown that 50% of the wild bird fecal samples, collected during winter, were contaminated with *C. jejuni* weather none of the samples collected in summer months were contaminated (Craven et al., 2000).

Temperature is correlated with *Campylobacter* spp., colonization of broilers in a study by Jonsson et al. (2012). Daily mean temperatures, above 6°C, had an important role in the colonization with a more significant effect accentuated by increasing temperatures. The same study also showed that below 0°C reduces the probability for a chicken flock to be positive for *Campylobacter*. *Campylobacter* spp. are known as sensitive to low temperatures which could explain the incremental effect caused by the increase in environmental temperatures.

## Water

*Campylobacter* has a high survival rate in water and thus can contaminate water reservoirs following translocation from pastures of grazing animals. Water chlorination appears to be very effective against *Campylobacter* (Newell and Fearnley, 2003; Hutchison et al., 2004). Only 18.8% campylobacters strains isolated from surface water in Louxembourg can be attributed to poultry, 61% to wild birds and 20.2% to other farm animals (Mughini-Gras et al., 2016). Spreading of animal feces on land was not correlated to increased presence of Campylobacter in surface waters (Sterk et al., 2016). Contamination of water in the broiler house usually follows colonization of a flock indicating that this is caused by contamination of water lines with microorganisms excreted from the birds. Water treatments need to consider the resistance of water borne protozoa such as *Tetrahymena pyriformis* which act as reservoirs for *C. jejuni* (Newell et al., 2011). The addition of organic acids has been examined and observations suggest that these provide partial effect in *Campylobacter* colonization and transmission. They can be used as part of ahurdle approach in conjunction with other measures (Sahin et al., 2015).

Drinker systems with nipples without cups were shown to be better than nipples with cups and bells in reducing the presence of *Campylobacter* in both Danish and Norwegian broiler flocks. *Campylobacter* may be introduced into the reservoirs of drinker systems with cups and bells via colonized birds or their droppings, allowing the water to become a dissemination vehicle (Borck Hog et al., 2016). Using municipal sources of water was deemed to be the best as opposed to using wells or boreholes. Private water supply was significantly linked to higher risk of *Campylobacter* contamination compared to the public water supply (Jonsson et al., 2012), however, this was not always the case (Hald et al., 2000).

## Vertical Transmission and Lag Phase

Most of the investigations to date have focused on horizontal transmission and farm management practices have been the primary focus of investigations aimed at finding solutions to reduce *Campylobacter*. There is sufficient evidence to support the vertical transmission of *Salmonella* (which can be transmitted via the eggs) however in the case of *Campylobacter* this evidence is absent (Newell and Fearnley, 2003). This statement is supported by the lack of *Campylobacter* colonization during the first weeks in the life of broilers hatched form eggs originated from breeder flocks infected with *Campylobacter.* This is further supported by the different genotypes in broilers in comparison to breeders that the eggs have been hatched from. However, *C. jejuni* can penetrate egg shells, indicating that contact with fecal material could contaminate the shells (Allen and Griffiths, 2001). Research has shown that *C. jejuni* can colonize egg contents by both oviduct colonization and fecal contamination of egg shells (Cox et al., 2012).

Further research is required to understand vertical transmission risks and measures required to reduce these risks throughout the supply chain. Chicks identified as negative on hatching remain negative until the lag phase (around 10 days of age). It is thought that there are other protective factors such as immunity that protect the chicks up to this stage. Further investigation into the physiological changes of the birds and the effect of changes in farm practices such as changes in feed composition, is required during this lag phase (Newell et al., 2011). Berndtson et al. (1996) have shown that *Campylobacter* are not detected in samples from newly hatched, 1 week old chicks or their drinkers. Artificial infection of 1 day old chicks with an invasive strain can cause diarrhea within 24 h (Berndtson et al., 1996). As described above the appearances of gastrointestinal disorders are associated with the age of the host as infection at 3 days of age with 10^9^ organisms failed to produce any detectable clinical change. This has also been confirmed in a longitudinal study showing the effect of age on colonization. The risk of flock infection increased with age. Several reports suggest that the immune support in the first 2 weeks plays a part in this. The environmentally stressed campylobacters are poor colonizers and they normally require an *in vivo* passage to enhance their colonization potential (Evans and Sayers, 2000).

Samples collected from the environment, air, feed, water, meconium and the fecal pools, of 2 and 7 old chickens, have always been identified to be negative by Damjanova et al. (2011). In contrast all flocks became colonized by *Campylobacter* between the third and sixth week of rearing. All finished flocks were found to be colonized.

## Lower Risk Factors

Feed is not seen as a high-risk *Campylobacter* contaminant within the broiler house. This is because the low water activity of the dry feed does not permit *Campylobacter* survival. The feed however can be a vehicle for horizontal transmission into the broiler house (Silva et al., 2011). Hald et al. (2000) showed that the incidence of Campylobacter was lower in farms that fed home grown wheat compared to farms that are depended of external supplies (Hald et al., 2000).

Chickens are known to ingest litter and research has shown that a significant of litter is consumed form the floor by the animals (Svihus et al., 2009). A study Torok et al. (2009) has shown that the caecal microbiota of chickens farmed on reused litter differed from that of chickens farmed on any of the other litter materials. Fresh litter is deemed to be low risk due to the low moisture content (Newell et al., 2011). In recent studies to show a controlled comparison, 60% of chickens in reused litter were positive for *Campylobacter*, compared to 33% in fresh litter. This observation suggests that used litter can act as a pool and source of *Campylobacter* (Sahin et al., 2015).

## Conclusion

The understanding of epidemiology and ecology of *Campylobacter* in poultry has been significantly improved over the past years. A minimum of 40 cells can constitute a successful infectious dose in chickens (Chen et al., 2006). Horizontal transmission is considered the main source of *Campylobacter* infection in poultry. *Campylobacter* are ubiquitous in the environment and can be transferred into the poultry farm in several different ways. The potential role of climatic factors as well as routine flock management practices have also to be taken under consideration. Increased biosecurity to minimize *Campylobacter* contamination should be of paramount importance during the summer period and when chicken flocks are thinned. The high prevalence of *Campylobacter* positive flocks and human cases of campylobacteriosis suggest that current biosecurity procedures are inadequate in ensuring *Campylobacter* negative flocks. Pre-harvest control measures in farms can help reduce *Campylobacter* dissemination in the environment, on farms and the food chain. Since no single contamination vehicle or route is responsible for *Campylobacter* contamination the different vehicles or routes should be tackled simultaneously. An integrated system that incorporates multiple pre- and post-harvest interventions as well as flock management interventions is necessary in order to reduce the risk from *Campylobacter* infections linked to consumption and/or handling of contaminated poultry meat. Maintaining such control measures on the farm can be difficult and it should be reinforced with farm worker education and incentives.

## Author Contributions

NS, AM, AR, SR, TC, AS, OG, and NC wrote the paper. All authors read and approved the final manuscript.

## Conflict of Interest Statement

The authors declare that the research was conducted in the absence of any commercial or financial relationships that could be construed as a potential conflict of interest.
